# Molecular Mechanisms Associated with ROS-Dependent Angiogenesis in Lower Extremity Artery Disease

**DOI:** 10.3390/antiox10050735

**Published:** 2021-05-07

**Authors:** Greg Hutchings, Łukasz Kruszyna, Mariusz J. Nawrocki, Ewa Strauss, Rut Bryl, Julia Spaczyńska, Bartłomiej Perek, Marek Jemielity, Paul Mozdziak, Bartosz Kempisty, Michał Nowicki, Zbigniew Krasiński

**Affiliations:** 1 The School of Medicine, Medical Sciences and Nutrition, Aberdeen University, Aberdeen AB25 2ZD, UK; g.hutchings.16@abdn.ac.uk; 2 Department of Anatomy, Poznan University of Medical Sciences, 60-781 Poznan, Poland; mjnawrocki@ump.edu.pl (M.J.N.); rutbryl@gmail.com (R.B.); julaspaczynska@gmail.com (J.S.); 3 Department of Vascular and Endovascular Surgery, Angiology and Phlebology, Poznan University of Medical Sciences, 60-848 Poznan, Poland; lukaszkruszyna@ump.edu.pl (Ł.K.); estrauss@ump.edu.pl (E.S.); zkrasinski@ump.edu.pl (Z.K.); 4 Institute of Human Genetics, Polish Academy of Sciences, 60-479 Poznan, Poland; 5 Department of Cardiac Surgery and Transplantology, Poznan University of Medical Sciences, 61-848 Poznan, Poland; bperek@ump.edu.pl (B.P.); kardiock@ump.edu.pl (M.J.); 6 Physiology Graduate Program, North Carolina State University, Raleigh, NC 27695, USA; pemozdzi@ncsu.edu; 7 Prestage Department of Poultry Science, North Carolina State University, Raleigh, NC 27695, USA; mnowicki@ump.edu.pl; 8 Department of Histology and Embryology, Poznan University of Medical Sciences, 60-781 Poznan, Poland; 9 Department of Veterinary Surgery, Institute of Veterinary Medicine, Nicolaus Copernicus University in Toruń, 87-100 Toruń, Poland

**Keywords:** peripheral arterial diseases, angiogenesis, neovascularization, atherosclerosis

## Abstract

Currently, atherosclerosis, which affects the vascular bed of all vital organs and tissues, is considered as a leading cause of death. Most commonly, atherosclerosis involves coronary and peripheral arteries, which results in acute (e.g., myocardial infarction, lower extremities ischemia) or chronic (persistent ischemia leading to severe heart failure) consequences. All of them have a marked unfavorable impact on the quality of life and are associated with increased mortality and morbidity in human populations. Lower extremity artery disease (LEAD, also defined as peripheral artery disease, PAD) refers to atherosclerotic occlusive disease of the lower extremities, where partial or complete obstruction of peripheral arteries is observed. Decreased perfusion can result in ischemic pain, non-healing wounds, and ischemic ulcers, and significantly reduce the quality of life. However, the progressive atherosclerotic changes cause stimulation of tissue response processes, like vessel wall remodeling and neovascularization. These mechanisms of adapting the vascular network to pathological conditions seem to play a key role in reducing the impact of the changes limiting the flow of blood. Neovascularization as a response to ischemia induces sprouting and expansion of the endothelium to repair and grow the vessels of the circulatory system. Neovascularization consists of three different biological processes: vasculogenesis, angiogenesis, and arteriogenesis. Both molecular and environmental factors that may affect the process of development and growth of blood vessels were analyzed. Particular attention was paid to the changes taking place during LEAD. It is important to consider the molecular mechanisms underpinning vessel growth. These mechanisms will also be examined in the context of diseases commonly affecting blood vessel function, or those treatable in part by manipulation of angiogenesis. Furthermore, it may be possible to induce the process of blood vessel development and growth to treat peripheral vascular disease and wound healing. Reactive oxygen species (ROS) play an important role in regulation of essential cellular signaling pathways such as cell differentiation, proliferation, migration and apoptosis. With regard to the repair processes taking place during diseases such as LEAD, prospective therapeutic methods have been described that could significantly improve the treatment of vessel diseases in the future. Summarizing, regenerative medicine holds the potential to transform the therapeutic methods in heart and vessel diseases treatment.

## 1. Introduction

The vascular network is essential for delivery of oxygen and nutrients, as well as removal of carbon dioxide and metabolic waste products from different tissues. In addition, messenger molecules such as hormones and growth factors are circulated in the bloodstream. The impairment of blood flow throughout the body can have devastating consequences. The body must have mechanisms to circumvent impairment or injury to the network. The expansion of this network in response to low availability of nutrients or oxygen is known as angiogenesis, a vital process regulated by signaling molecules such as vascular endothelial growth factor (VEGF). Specifically, in the absence of cellular oxygen, hypoxia-inducible factors (HIFs) are activated and translocated to the nucleus where they upregulate genes including VEGFA [1,2]. Through signaling by VEGF, mature endothelial cells (ECs) are directed to sites of hypoxia to participate in formation of new blood vessels.

Angiogenesis, while vital for wound repair, can be pathogenic in the context of cancer, as development of vessels around the tumor provides sustenance, and a vehicle for metastasis via the circulatory system. Strict control of angiogenesis is desirable to prevent metastasis, potentially achieved through balancing of angiogenic growth factors and angiogenic inhibitors as treatments. Mixed results in using growth factors as angiogenic treatments have resulted in modern alternatives such as cell-based therapies and hydrogels as delivery mechanisms.

Arteriogenesis, as distinct from angiogenesis, refers to the development of collateral vessels from arterioles to restore circulation through development and expansion of the diameter to create new arteries, while angiogenesis describes the expansion of existing capillary density to reach ischemic sites [3,4]. Lymphangiogenesis describes a similar process of repair and expansion in the lymphatic system, while vasculogenesis refers to the differentiation of angioblast precursors into endothelial cells, and subsequent establishment of primitive vessels and vessel networks [2]. Angiogenesis, vasculogenesis, lymphangiogenesis and arteriogenesis, collectively referred to as neovascularization, are vital processes to tissue regeneration and repair. Typical stimuli for neovascularization include hypoxia, and inflammation. In response, cells will release a variety of growth factors, which will initialize neovascularization. Endothelial cells, in response to these factors, will dissociate, migrate and proliferate, by budding and sprouting, to establish new vessel structures.

Stem cell therapies show promise as angiogenic treatments in diseases affecting the circulatory system. Specifically, the secretome of mesenchymal stromal cells (MSCs), as well as that of adipose-derived stem cells (ASCs), is known to contain pro-angiogenic miRNAs and growth factors, which can induce angiogenesis to enhance wound healing, cardiomyopathies and peripheral artery disease.

## 2. Peripheral Arterial Disease

Peripheral arterial disease (PAD, other terms used for this condition are peripheral vascular disease, peripheral arterial occlusive disease, and lower extremity arterial disease (LEAD)) defines all arterial diseases other than coronary arteries and the aorta. It is a global health problem affecting over 40 million individuals in Europe [5]. The most common cause of PAD is atherosclerotic vascular disease. Symptoms of PAD most often result from progressive arterial narrowing due to ongoing atherogenesis, which restricts blood flow. PAD is an international health problem, and patients with PAD have an increased risk of mortality compared to the general population. The main cause of death in patients with PAD is cardiovascular (CV) events [6]. Atherosclerosis is a progressive disease related to age, smoking history, and diabetes that may simultaneously affect multiple arteries [7].

There are approximately 202 million people affected with lower extremity artery disease (LEAD) worldwide [5]. LEAD usually appears after the age of 50 years, with an increase after the age of 65 years to as high as 20% in people over the age of 70 [8]. In symptomatic patients with LEAD, the most typical presentation of reduced blood flow is intermittent claudication (IC), thigh or calf pain with walking due to temporary ischemia of the leg muscles during exertion. The clinical manifestation of LEAD is heterogenous. Symptoms of ischemia depend on the degree of stenosis and insufficiency of blood supply to the distal tissue. Chronic limb-threatening ischemia (CLTI) is defined by the presence of ischemic rest pain, with or without tissue loss (ulcers, gangrene) or infection [7]. The natural history of a patient with either IC or CLTI is associated with considerable mortality, impaired functional capacity, and low quality of life.

Low perfusion can result in ischemic pain, non-healing wounds, and ischemic ulcers. In clinical practice, strategies for the treatment of LEAD focus on the reduction of the risk factors of atherosclerosis and revascularization procedure, which may be either open bypass surgery or endovascular recanalization. There is also a clear evidence that supervised exercise therapy improves lower limb symptoms and quality of life among LEAD patients. Supervised physical exercise (PE), a natural stimulus for vascular remodelling, plays a crucial role in the pathophysiology of PAD both in the prevention and treatment [8].

**Figure 1:**
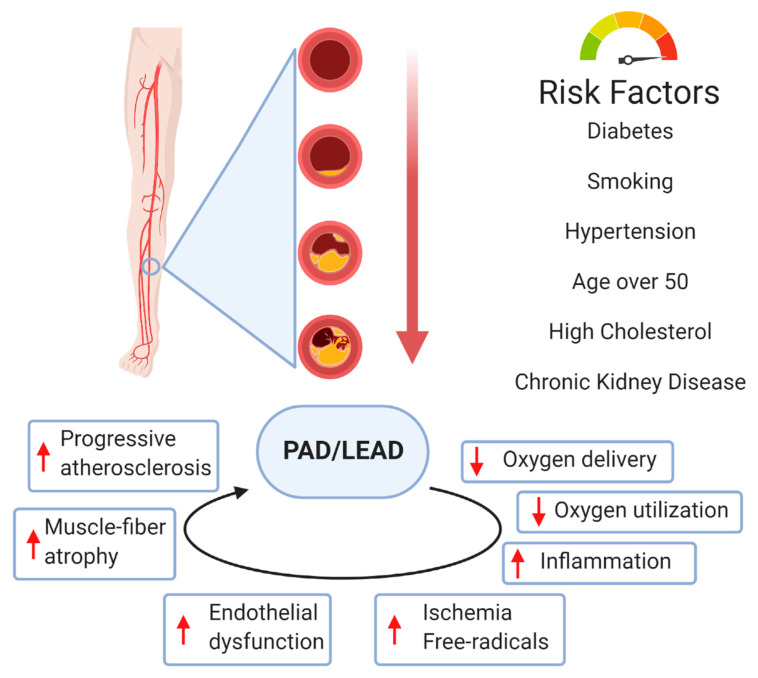
The main risk factors for the progression of peripheral artery disease (PAD)/lower extremity artery disease (LEAD) and mechanisms of pathophysiological impairment associated with PAD. Created with BioRender.

Smoking is associated with peripheral arterial disease (PAD), and the risk increases with smoking intensity [9]. Diabetes is strongly associated with LEAD, with ORs ranging from 1.9 to 4 in population studies (Figure 1) [10]. The prognosis of LEAD in diabetic patients is poorer than in non-diabetic patients, with a 5-fold increased risk of amputation, a specific pattern more frequently affecting distal arteries, frequent coexistence of neuropathy, and a higher risk for infection [11]. Inflammation is involved in atherosclerosis pathophysiology. Several markers of inflammation (e.g., high-sensitivity C-reactive protein, fibrinogen, interleukin 6) are associated with an increased risk of the presence, progression and complications of LEAD [12,13].

## 3. Molecular Basis of Vessel Disease

LEAD usually involves atherosclerotic disease in the abdominal aorta, iliac, and femoral arteries. The pathophysiology of atherosclerotic plaque involves complex interactions between cholesterol metabolism and vascular cell activity. It is also known that disturbance of the laminar arterial flow in PAD plays an essential role in the adhesion of inflammatory blood cells to the arterial wall and plays a role in plaque generation [14]. The hemodynamic consequences of atherosclerotic plaque depend on the degree of stenosis/occlusion. A narrowed vessel progresses towards chronic total occlusion. LEAD patients demonstrate high grades of inflammation and active oxidative stress, which are vital mechanisms in PAD pathophysiology [14]. There is an interest in the vaso-protective effects of heme oxygenase 1 (HO-1) as a potential antioxidant by affecting the proliferation, migration, and adhesion of smooth vascular muscle cells, endothelial cells, and leukocytes [15]. It was previously suggested that PE acts on endothelial functions by regulating genes involved in modulating oxidative metabolism, cell apoptosis, cell growth and proliferation, and endothelial vascular nitric oxide synthase (eNOS) [14,16].

Vessel wall remodelling and angiogenesis in peripheral arteries appear to be the most important tissue response process to the atherosclerotic injuries [17]. Extracellular matrix (ECM) provides a mechanical scaffold and support to cell migration regulated by cytokines, growth factors, and enzymes, such as matrix metalloproteinases (MMPs) [18,19]. Hernandez-Aguilera et al. suggested that atherosclerosis of the lower extremities is due to an excessive reparative response, which includes factors favoring ECM degradation [19,20]. Decreased levels of structural proteins of the arterial wall are highly influenced by MMP activity [19]. These proteins also have defined roles in maintaining normal function and migration of smooth muscle cells [21]. Connective tissue turnover in the structural and signaling properties of the arterial cells plays a central role in the LEAD development [22]. Single nucleotide polymorphisms of the genes encoding for some MMPs have also been associated with the risk of developing LEAD. Blankenberg et al. [23] reported a study that focused on two MMP-9 gene polymorphisms: the MMP-9/C-1562T promoter polymorphism, which influences the transcriptional activity of the MMP-9 gene, and the exonic MMP-9/R279Q polymorphism, which leads to an amino acid exchange in the catalytic domain of MMP-9. The authors described a significant association between the R279Q polymorphism and cardiovascular events in patients with stable angina.

Arterial calcification was previously considered a passive degenerative process but is now recognized as a complex process actively regulated by several cell molecules [24]. Arterial calcification mainly involves the intima and media, and is associated with cardiovascular risk factors, such as diabetes, hypertension, hyperlipidemia, and chronic kidney disease [25]. Arterial calcification is related to the apoptosis of vascular smooth muscle cells and macrophages [26]. Studies provided by Hui laboratory [27] focused on the genetic variants of the matrix Gla protein (MGP), a protein known as a key player in in vivo inhibition of calcification. The authors analyzed rs4236, rs1800801, and rs1800802 variants of the *MGP* gene, and showed association with calcification on the arterial wall but not with calcification in atherosclerotic plaques [27]. Other studies showed that different types of arterial calcification develop through various molecular mechanisms in different vessel types [28]. In contrast to carotid and coronary arteries, arterial calcification of LEAD is mostly located in the media.

Several genotypes serve as potential risk factors for atherosclerosis. However, evidence of their clinical relevance is weak. Some of the most common risk factors for PAD (diabetes, dyslipidemia) are heritable. However, PAD may also result from genetic factors acting independently. Identification of such genes may provide insights into pathophysiologic mechanisms of PAD progress and facilitate the development of novel therapeutic approaches [29]. In contrast to coronary artery disease, relatively few genetic variants that influence susceptibility to PAD have been discovered because there may be more significant clinical and genetic heterogeneity in PAD patients. Definitively, genetic factors may have an impact on the early onset of PAD in young adults, including these mechanisms affecting the process of inflammation, thrombosis, and the metabolism of cholesterol and homocysteine. Among these genetic disorders, familial hypercholesterolemia or hyperhomocysteinemia are the most known. For example, Flex et al. [30] explored the association between the interleukin-6 gene (IL-6)-174 G/C single nucleotide polymorphism (SNP) and the risk of peripheral artery occlusive disease. The authors concluded that the analyzed polymorphism is important in the pathophysiology of ischemic diseases of the lower extremity [30]. Several other candidate genes involved in the process of atherosclerosis and regulating inflammation and coagulation pathways and vascular matrix regulation were also analyzed, such as β-fibrinogen, eNOS, MTHFR, and glutathione S-transferase [31]. However, any reported associations between variants in these genes and PAD have not been confirmed [29].

### 3.1. Neovascularization

In healthy individuals, reducing blood flow to the lower extremity is followed by a physiological process to limit ischemia in the distal tissue by expanding the vasculature. Neovascularization is regulated by mechanisms that respond to ischemia, hypoxia, or shear stress [32]. Neovascularization is the process of blood vessel development and growth through three distinct biological processes: vasculogenesis, angiogenesis, and arteriogenesis [4]. Two forms of neovascularization, angiogenesis, and arteriogenesis can be distinguished during LEAD. Physiological studies supported the hypothesis that after ischemia, the neovascularization process can re-establish blood flow through the ischemic tissue and protect the extremities from blood flow loss. It is important to note that compromised arterial inflow results in angiogenesis due to distal tissue ischemia and arteriogenesis that occurs close to the site of arterial trunk occlusion [32]. Structural and functional adaptations of the vasculature can also be induced by exercise training in humans [33]. LEAD symptoms may be the result of not only an obstructive vascular process but also an impaired neovascularization response [1].

### 3.2. Vasculogenesis

During embryonal development, blood vessels first appear due to vasculogenesis, the formation of capillaries from endothelial cells differentiating in situ from groups of mesodermal cells [2]. During embryonal life, microvascular collaterals develop after the arterial trees. These collaterals increase their diameter and wall thickness as a consequence of the growth of a muscular layer [4,34]. Hemodynamic forces regulate the differentiation of arteries and veins from the primary vascular plexus. It was suggested that vessels adapt to flow during arteriogenesis [4]. Other observations have indicated that vasculogenesis is not limited to early embryogenesis but also has a physiological role during vascular disease in adults. Postnatal vasculogenesis was explained by the presence of circulating endothelial cells and endothelial progenitor cells in human and several mechanisms of blood vessel formation during tumor growth [4,35].

### 3.3. Angiogenesis

Angiogenesis is the process of new capillary formation from pre-existing capillary beds that involves proliferation, sprouting, and migration of endothelial cell migration. Angiogenesis occurs naturally during wound healing, tissue growth, and repair. The process of angiogenesis is highly controlled, dependent on a balance of both pro-angiogenic and anti-angiogenic factors. The process of angiogenesis results from complex interactions between growth factors, endothelial cells, pericytes, fibroblasts, smooth muscle cells, and the extracellular matrix. As a result of these interactions, extracellular proteolysis, endothelial cell migration, proliferation, and differentiation, and finally, vascular wall remodeling can occur [4]. Vascular endothelial growth factor (VEGF) is the main factor stimulating angiogenesis in response to tissue hypoxia [36]. Angiogenesis is primarily stimulated by tissue hypoxia via activation of hypoxia inducible factor (HIF). Specifically, in the absence of cellular oxygen, hypoxia-inducible factors (HIFs) are activated and translocated to the nucleus where they upregulate genes including VEGFA, angiopoietins, and nitric oxide [1,2,37]. Through signaling by VEGF, mature endothelial cells (ECs) are directed to sites of hypoxia to participate in the formation of new blood vessels. The endothelial cell activation is associated with cytokine release, initiation of vasodilation, and increased endothelial cell permeability. VEGF promotes the release of many proteolytic factors, such as matrix metalloproteases, which degrade the extracellular matrix and facilitate endothelial cell migration [38]. Once a functional vascular network is formed, the new vessels are remodeled to become a mature vessel system [32]. Angiogenesis describes the vascular tissue remodeling and is characterized by the expansion of existing capillary density to reach ischemic sites, maintaining the lower extremity function in patients with LEAD.

### 3.4. Arteriogenesis

As a distinct process from angiogenesis, arteriogenesis refers to the growth of new arteries and arterioles either de novo or from pre-existing arterial collaterals [3,39]. It mainly involves the proliferation of vascular endothelial cells (ECs) and smooth muscle cells (SMCs) [40]. As a result of the occlusion of the main arterial trunk, the pre-existing arterio-arteriolar anastomoses between interconnected perfusion territories can undergo adaptive enlargement that develops into a functional network of collateral arteries [32]. Arteriogenesis is critical to the restoration of tissue perfusion following the development of a functionally significant decrease of arterial inflow during LEAD [41]. The transformation of native microvascular collateral arterioles into functional arteries with consequent recovery of blood flow is particularly observed after ischemic injury [42].

It was previously suggested that vessel segments could adapt to the amount of flow [43]. Collateral growth is driven by hemodynamic forces and leads to initial vasodilation due to increased levels of nitric oxide [44]. While hypoxia is the primary driver of angiogenesis, arteriogenesis is mainly induced by a combination of shear stress and other mechanical factors [36,39,41]. Pulsatile shear stress activates the cascade of events that leads to the development of collateral circulation [4,45]. Several genes are controlled by shear stress-responsive elements in their promotor, and fluid shear stress influences these genes’ expression. The role of mechano-sensors and transducers that convey the shear stress message during collateral remodeling has been suggested as a mechanism directing neovascularization [46,47,48]. A fluid shear stress-associated transient receptor potential cation channel, subfamily V, member 4 (trpv4), turned out to be upregulated transiently after endurance training [49,50]. Shear stress increases the expression of an isoform of connexin, connexin-37, in endothelial cells [51].

Arteriogenesis consist of two phases: the early inflammation phase and the later phase of vessel diameter increase, remodeling, and maturation. Hemodynamic forces in the collateral vessels are the primary stimulus to initiate arteriogenesis. As a result of the increased shear stress force, endothelial cells express monocyte adhesion molecules: platelet endothelial cell adhesion molecule (PECAM-1), MCP-1, intracellular adhesion molecule (ICAM-1), and vascular cell adhesion molecule (VCAM-1) [52]. Endothelial cells regulate adhesion molecule gene expression through a mechano-transduction process by specific shear stress receptors [32]. Cytokines and cell adhesion molecules attract monocytes to adhere and invade the vascular wall. Recruited monocytes infiltrate the vessel wall and transform into macrophages. Once activated, they produce TNF-α and attract more monocytes. Recruitment of circulating monocytes and resident macrophages promotes arteriogenesis by their ability to secrete metalloproteinases, chemokines, and growth factors [53]. The proliferation of the endothelium is followed by smooth muscle proliferation and migration to form a new neointima. Little is known about the mechanisms triggering SMC proliferation in arteriogenesis. Growth factors, such as fibroblast growth factor 2 (FGF-2) and platelet-derived growth factor-BB (PDGF-BB), are essential for SMC proliferation in arteriogenesis [54]. Finally, enlargement of the blood vessel occurs by remodeling the adventitia through fibroblast activation and proliferation. These events lead to new collateral vessel development in postnatal life.

Arteriogenesis differs from angiogenesis in several aspects, the most important being the dependence of angiogenesis on hypoxia and the dependence of arteriogenesis on inflammation. Arteriogenesis occurs in tissues near arterial stenosis/occlusion, whereas peripheral ischemic regions undergo angiogenesis, which is the growth of new capillaries [55]. Collateral vessels resulting from arteriogenesis are typically surrounded by normoxic tissue [56]. While molecular regulation of angiogenesis is well analyzed, events regulating arteriogenesis are still controversial. As well as for angiogenesis, VEGF is also critical to arteriogenesis [57]. In arteriogenesis, VEGF activation of extracellular signal-regulating kinase 1/2 (ERK1/2) induces endothelial cell proliferation, network formation, and increased lumen size. Disruption of VEGF-induced endothelial ERK1/2 signaling results in decreased arteriogenesis [58]. The activation of this signaling is modulated by NRP-1 [59]. VEGF, TGF-α, and FGF-2 stimulate the proliferation of endothelial cells, whereas TGF-β, GM-CSF, monocyte chemoattractant protein-1 (MCP-1), and PDGF stimulate arteriogenesis through the proliferation of smooth muscle cells [60]. The proliferation of endothelial cells and smooth muscle cells leads to lumen size expansion of the collateral artery [61]. In contrast to angiogenesis, arteriogenesis requires a coordinated response that involves multiple cell types, not only endothelial and vascular smooth muscle cells [41]. Several inflammatory cells, including lymphocytes, natural killer (NK) cells, macrophages, and mast cells, were also suggested to play a role in arteriogenesis [62,63,64]. The presence of inflammatory cells is critical as these cells serve as the major source of VEGF in the absence of tissue ischemia [41,65,66]. Mast cells have also been associated with arteriogenesis and collateral formation [67,68]. The proinflammatory response during arteriogenesis is beneficial in restoring blood flow, but may lead to enhanced progression of atherosclerosis [68].

Collateral circulation occurs between arterioles in most healthy tissues and in pathological conditions like ischemic injury [39]. Arteriogenesis can be induced by regular exercise or muscle loading in humans in physiological conditions [33,49,69]. A more regular exercise regimen was also proposed to be required for sufficient arteriogenesis to compensate for an arterial occlusion in LEAD [49]. It explains why significant stenosis/occlusion of main arteries may remain asymptomatic in patients with chronic LEAD for many years. Exercise therapy can influence the vascular system supply to the ischemic limb by promoting both angiogenesis and arteriogenesis [33]. An increase of angiogenic and arteriogenic factors, such as VEGF and HIF-1 alpha, was observed after regular exercise [70]. Additionally, higher concentrations of heat shock proteins and the enzyme nitric oxide synthase (NOS) in blood during exercise therapy was noticed [71]. Nitric oxide (NO) is an important cellular signaling molecule produced in high levels in muscle by neuronal NOS [33]. Under ischemic conditions, the role of NO in vasodilatation is increased compared with non-ischemic conditions, which results in an up-regulation of endothelial NOS (eNOS) [72]. Regular exercise training in the patient with LEAD leads to similar mechanisms of vascular adaptation such as increased fluid shear stress and promotes arteriogenesis. After successful revascularization, collateral arteries shrink or disappear as the main blood flow is directed back through the re-opened artery or vascular reconstruction. Supervised exercise therapy is considered to be a potential therapeutic option in chronic LEAD patients under conservative treatment.

## 4. Tissue Regeneration and Repair by Neovascularization

### 4.1. Hypoxia

**Figure 2:**
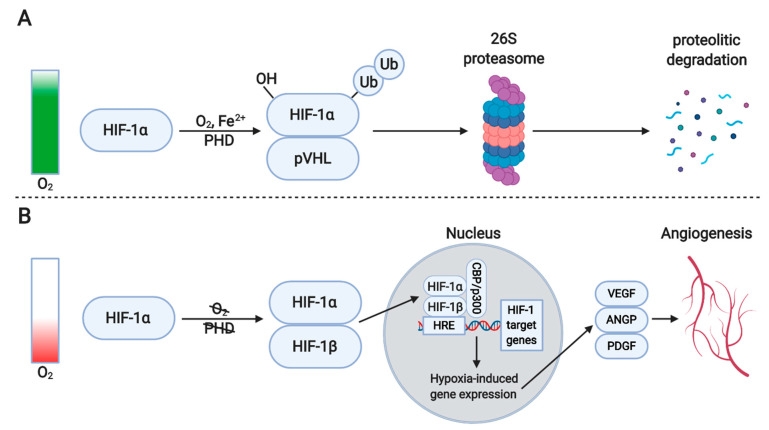
The regulation of hypoxia-inducible factor 1α (HIF-1α) activity. (**A**) Under normoxic conditions, prolyl hydroxylase (PHD), in the presence of Fe^2+^ and an oxygen molecule, hydroxylates proline residues in the oxygen-dependent degradation domain of HIF-1α. This allows recognition and interaction with the von Hippel-Lindau (pVHL) E3 ubiquitin ligase leading to HIF-1α ubiquitination and subsequent degradation by 26S-proteasome. (**B**) Under hypoxic conditions, the hydroxylation and degradation of HIF-1α are blocked. Thus, HIF-1α can dimerize with HIF-1β, enter the nucleus and bind hypoxia response elements (HREs) within the promoters of target genes. Together with the co-activator proteins p300 and CBP, the HIF complex transcriptionally regulates the expression of its target genes such as VEGF, PDGF and ANGP, finally regulating many processes including angiogenesis. Created with BioRender.

As oxygen homeostasis is vital to an organism’s survival, complex cellular processes exist to adapt to pathological hypoxic conditions. Under normal oxygen levels, hypoxia-inducible factor (HIF) proteins are marked for ubiquitination by prolyl hydroxylase domain enzymes (PHD), a class of oxygen-sensitive enzymes, and thus kept inactive. When oxygen is unavailable and a cell undergoes a state of hypoxia, the inactivity of PHDs allows HIFs to enter the nucleus and activate expression of angiogenic factors. Among the target genes activated is VEGF-A, the principal regulatory factor directing angiogenesis (Figure 2). Additionally, 2% of all genes in endothelial cells of the arteries are indirectly regulated by HIF-1, and expression of at least 40 genes is directly controlled by this molecule, including angiopoietin-2 (ANGP2) [73,74]. While HIF-1α is expressed in all nucleated cells, HIF-2α is specific to the endothelium [74]. As well as VEGF, erythropoietin (EPO) is also upregulated during states of hypoxia, stimulating an increase in red blood cell count. Hypoxia drives angiogenesis, although inflammation and hemodynamic factors are more important stimuli for arteriogenesis [75].

### 4.2. Inflammation

Upon injury or infection, inflammatory mediators released by mast cells, as well as other resident immune cells, recruit macrophages and T-lymphocytes to the pathogenic site. These cells act as a source of angiogenic and arteriogenic factors including VEGF, bFGF and TGF-β [75]. Recruitment of endothelial cells, smooth muscle cells, leukocytes and other cell types helps establish and stabilize the neovasculature. In this way, inflammation and angiogenesis are interdependent and highly linked processes in ischemia and other pathologies including tumor formation. Tumor cells synergize with nearby immune cells to establish neovascularization in the surrounding tissue [76].

**Figure 3:**
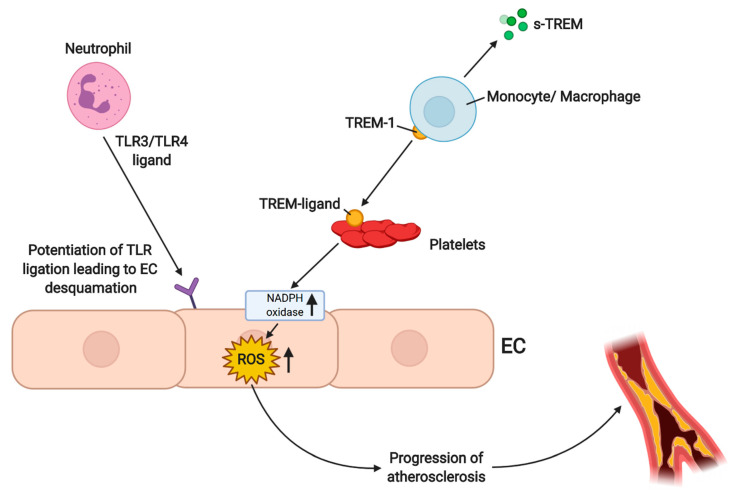
Neutrophils and monocytes/macrophages in peripheral artery disease (PAD). Neutrophils enhance the activation of monocytes via toll-like receptors (TLRs). TLRs are pattern recognition proteins, and their agonism leads to multiple important cellular mechanisms, like the generation of endoplasmic reticulum stress-ROS, and impairment of endothelial cell repair. Neutrophils potentiate TLR ligation leading to endothelial cell detachment and plaque erosion. Another mechanism indicating the role of neutrophils and monocytes/macrophages in oxidative stress shows that triggering receptors expressed on myeloid cells-1 (TREM-1), as an example of an inflammation marker, participate in the stimulation of these cells via interaction of TREM-ligand with TREM-1, which leads to an increased production of reactive oxygen species (ROS). Created with BioRender.

Interestingly, the presence of neutrophils and macrophages is itself enough of a stimulus to initiate neovascularization at a non-ischemic site (Figure 3) [75]. Macrophage promotion of angiogenesis was shown to be linked to uptake of free hemoglobin [77]. An inability to recruit macrophages into areas of neovascular response will impair vessel growth, suggesting that inflammation and recruitment of cells of the immune system are intrinsic to neovascularization [75].

### 4.3. ROS-Dependent Angiogenesis in PAD/LEAD

ROS can accumulate through exposure to chemical agents or radiation in the environment but are also generated as a product of normal metabolism. Although high levels of ROS are damaging to tissues, at lower levels they function as necessary signaling molecules for processes including angiogenesis in arterial diseases [78]. In PAD and LEAD, dysfunction of blood vessels leads to highly hypoxic environments, in which surrounding cells will respond by upregulating NADPH oxidase (NOX). In blood vessels, NOX catalyzes the production of ROS in the endothelial cell membrane, which contributes to the initiation of angiogenesis [79]. In experiments on mice, NOX2 was shown to be upregulated in newly formed vessels following angiogenesis, and knockout mice missing NOX2 were deficient in wound healing [80]. Contrastingly, in a type-1 diabetic model, inhibition of NOX2 improved neovascularization in mice following injury [81]. More recently, lowering ROS levels by microRNA-210 overexpression was shown to increase wound healing in an experimental model of PAD [82]. Maintenance of the correct level of ROS at sites of injury is therefore essential for appropriate cellular signaling in regeneration processes, and levels too low or high could be deleterious. NOX also plays a role in supporting newly formed vessels at sites of injury by upregulating proliferation of vascular smooth muscle cells. The production of ROS by NOX in response to growth factor signaling following injury therefore upregulates migration and tubule formation of endothelial cells, but also proliferation of supporting cells in new vessels [79].

### 4.4. Angiogenic and Arteriogenic Growth Factors

There are a number of important signaling molecules involved in neovascularization such as VEGF, FGF, ANGP, PDGF, TGF-β, IGF-1, SDF-1, MCP-1 and IL-8. Upon binding, specific receptors on the endothelial cell surface, intracellular signaling pathways regulating proliferation, migration and differentiation are triggered. The ‘angiogenesis hypothesis of ageing’ supports the idea that supplementation by some of these important growth factors may help combat lesser ailments in elderly people by encouraging neovascularization. Blood capillary density in the brain and muscles gradually lessens with age and the process has been linked to reduced levels of circulating angiogenic growth factors [83]. There have however been mixed results in studies evaluating effectiveness of angiogenic factors as therapies for coronary artery disease, angina and other ailments [75].

#### 4.4.1. VEGF

Vascular endothelial growth factor (VEGF) is secreted by hypoxic or expanding vessel tissues and is the main activating molecule of angiogenesis. VEGF-A is expressed by hypoxic cells to activate ECs via a paracrine mechanism, and it can also be expressed in ECs to act in angiogenesis [84]. The VEGFRs form a family of tyrosine kinase receptors. VEGFR-1, found on smooth muscle cell and monocyte plasma membranes, in addition to endothelial cells, has a high affinity for VEGF but does not seem to directly mediate migration or proliferation of ECs. VEGFR-2 is expressed on hematopoietic and EC precursor cell surfaces, mediating chemotaxis and EC mitogenesis, but has reduced expression in EC of the mature endothelium. VEGFR-3 is associated with the lymphatic endothelium and lymphangiogenesis [85].

Extracellular VEGF interacting with a VEGFR receptor on the endothelial cell surface induces a Dll/Notch signaling cascade, activating genes to initiate and co-ordinate angiogenesis. A study employing a murine model showed that Notch inhibition causes upregulation of VEGF-A in endothelial cells, and a subsequent increase in EC proliferation and sprouting [84]. VEGF is inhibited by endogenous molecules such as angiostatin and endostatin, which functionally oppose the actions of VEGF by promoting apoptosis and thereby negatively regulate angiogenesis [86]. Bleda et al. [87] compared *VEGF* gene variants in diabetic patients with different stages of vascular disease. Researchers analyzed insertion/deletion of −2549 (rs35569394), as well as −2578 C/A (rs699947) and +405 G/C (rs2010963), polymorphisms associated with different diabetic vascular complications. The authors found a higher frequency of +405 CC and −2578 CC genotypes in the claudication group, while the presence of +405 GG and −2578 AA genotypes was more common among critical limb ischemia patients. These results suggest that the +405 GG and −2578 AA genotypes of the VEGF gene are associated with the severe stage of LEAD [87].

The 2003 VIVA (Vascular endothelial growth factor in Ischemia for Vascular Angiogenesis) trial aimed to establish the efficacy and safety of intracoronary and intravenous infusions of recombinant human VEGF in patients with angina. Tests of efficacy included treadmill exercises, measurement of angina classification and quality of life tests. In high-dose VEGF patients, improvements were seen in all three areas measured by day 120, whereas there was minimal difference in results between placebo and low-dose patients [88]. More recently, there have been successful attempts to stimulate healing of ischemic sites in a mouse model by infusion of angiogenic factors via alginate nanogels. The study suggested that low-dose treatments of IGF together with VEGF could significantly improve angiogenesis at the ischemic site and could translate to humans to treat peripheral artery disease. The dosage of IGF accompanying VEGF offset the age-diminished response of VEGF treatment alone. This could be explained by age-related decreases in circulating IGF levels impacting angiogenic responses. Low levels of circulating IGF in old age have also been linked to low capillary density [89].

#### 4.4.2. FGF

Both acidic and basic fibroblast growth factors (aFGF and bFGF) are involved in inducing angiogenesis, stimulating EC proliferation, and migration. Intracellular growth factor concentration positively correlates with mitogenesis and subsequent EC proliferation and sprouting. However, when mitogenic stimulation reaches a saturation point, there is a negative effect on angiogenic proliferation, suggesting a dose dependent stimulatory or inhibitory effect [90]. bFGF, as well as acting as a mitogen of endothelial cells, can cause ECs to form tube-like structures in vitro using three-dimensional collagen matrices, a characteristic of developing vessels during angiogenesis. However, inducing FGF deficiencies in mice only mildly hindered angiogenesis in wound healing [91].

bFGF treatment in mice caused formation of capillary-like structures composed of fibrocytes, demonstrating an angiogenic fibrocyte phenotype and potentially an important role in angiogenesis [92]. Fibrocytes, a group of fibroblast-like cells derived from bone marrow, are known to play various important roles such as releasing a range of inflammatory cytokines, recruiting immune cells via expression of chemokine receptors and participating directly in tissue fibrosis. However, it is also possible that fibrocytes are involved in angiogenesis, both positively and negatively regulating the process by release of various growth factors such as VEGF, and angiogenic inhibitors [92].

FGF23, together with FGF19 and FGF21, unlike the classic FGFs, function as endocrine hormones and form a separate group in the FGF family [93]. FGF23 is a bone-derived hormone that plays an important role in the regulation of blood calcium level in cooperation with the protein Klotho, the cofactor implicated in the binding and activation of FGFRs by FGF23 [94]. The FGF23/Klotho axis appears to play a critical role in the pathogenesis of vascular disease, and analysis of polymorphisms in Klotho (like rs564481 or rs9536314) confirm this statement [95,96].

The 2002 FIRST (FGF Initiating RevaScularization Trial) study investigated the effects of intracoronary infusion of recombinant bFGF in human patients with coronary artery disease. A single intracoronary infusion resulted in symptomatic improvement in the short term only, which was negligible after 180 days, and did not include exercise tolerance tests, for which no improvement was recorded [97].

Combinations of growth factor treatments; FGF with VEGF, and FGF with platelet-derived growth factor-β (PDGF-β), have shown promise in improving vessel growth and formation of collateral networks in rat and rabbit models, suggesting that clinical trials investigating these factors as potential treatments should be investigated, but there have been results in previous studies [98,99].

The TAMARIS clinical trial published in 2010 investigated the effects of delivering FGF using a non-viral vector to patients with critical limb ischemia. Despite the known angiogenic properties of FGF, and a promising phase 1 and 2 trial, where ulcer severity and amputation risk were reduced following intramuscular administration of FGF, delivery was entirely ineffective in preventing risk of amputation or death during phase 3 [99]. Thus, although potential was shown for FGF as a treatment in vascular diseases, the failure to determine efficacy as a viable treatment in critical limb ischemia halted further human clinical trials [100]. However, bFGF has demonstrated therapeutic value in clinical trials where topical application significantly increased the healing of patients with pressure ulcers. Treated tissues showed an increase in wound closure, accompanied by capillary formation and an increase in the fibroblast population in wound tissue [101].

#### 4.4.3. Angiopoietin

The angiopoietins (ANGP), responsible for stability and support during angiogenesis are a family of four vascular growth factors that bind Tie receptors (specifically Tie2) on the endothelial cell surface. ANGP1, helps maintains the crucial EC barrier in vessels, but also plays an important role in EC survival, migration and sprouting [102]. Mouse studies have shown that deficiency of ANGP1 impairs vascular remodeling, causing death during embryonic development. Conversely, overexpression of ANGP1 caused more numerous blood vessels and branches of increased size to develop [85]. Investigations into the synergy of ANGP1 and VEGF in a mouse model concluded that both growth factors seemed to be additive in their angiogenic effects. Vascular leakage induced by overexpression of VEGF and/or inflammatory agents, was counteracted by the actions of ANGP1 [103]. ANGP2 works as an antagonist of ANGP1, binding to the Tie2 receptor in the vasculature, and may thus prevent excessive vessel growth and sprouting in vivo [85]. Additionally, ANGP1 but not ANGP2 is expressed in mural cells such as smooth muscle and pericytes, in addition to ECs. ANGP3 and ANGP4 are orthologs in the mouse and human respectively, known to interact with Tie2, although their regulatory functions in angiogenesis are not well understood [104].

#### 4.4.4. Non-Coding RNAs in Neovascularization

Micro-RNAs (miRNAs) are 21–23 base pair-long, non-coding nucleic acids, which significantly affect gene expression by interaction with complementary mRNA sequences to induce RNA silencing. The single-stranded miRNAs are thought to be expressed in all tissues and are involved in the regulation of physiological processes throughout the body, including angiogenesis and wound healing [105]. Specifically, miR-221/222 overexpression may inhibit angiogenesis by interfering with genes important to tube and capillary formation, presenting the possibility that inhibition of this miRNA may promote these processes [106]. miR-126 expression seems to be vital in wound healing, with knockout studies producing severely deleterious phenotypes in a mouse model [107]. It is proposed that miR-126 regulates expression of the anti-angiogenic factor SPRED-1, and thereby enhances the actions of VEGF and FGF to induce angiogenesis. In another study, miR-27b was shown to downregulate AMPK signaling, and that inhibition of this micro-RNA was sufficient to improve post-stroke recovery in a murine model by enhancing angiogenesis [108]. Additionally, key differentiation genes are known to be affected by miRNAs, representing new opportunities in regenerative medicine and control of cell fate [109]. miR-1 and 21 are both implicated in the formation of vascular smooth muscle cells, a process vital to angiogenesis [110]. As regulators of important physiological processes including neovascularization, micro-RNAs represent a valuable area of research in developing new targets for therapies in vessel-related diseases. Finally, as previously mentioned, miR-210 is an important inhibitor of ROS formation at sites of injury, strictly maintaining ROS levels to promote wound healing [82].

#### 4.4.5. Vasculogenesis and Lymphangiogenesis

Angioblasts are endothelial progenitor cells (EPCs) assemble to create primitive blood vessels during vasculogenesis. They originate in the mesoderm where FGFs act to stimulate differentiation. Although mainly active during embryonic development, vasculogenesis is a process also active during adult life, albeit rarely. Notch and ephrin proteins control differentiation pathways into vein and artery specific endothelial cells [111].

Shortly after the establishment of the vascular system by vasculogenesis and angiogenesis, lymphangiogenesis begins by budding and sprouting of ECs of the existing veins to develop the lymphatic system. The lymph system drains fluid, proteins, pathogens and migrating cells into the bloodstream. The lymph structure is characterized by an absence of smooth muscle/pericyte supporting cells, basement membrane and tight cell junctions. As a result, the walls of the endothelium are more permeable and can absorb larger molecules. The lymphatic system represents an important avenue for cancer metastasis [74].

Similar to VEGF-A and angiogenesis, VEGF-C controls migration and sprouting of lymphatic endothelial cells in developing vessels. ANGP2 and the master regulator of lymphangiogenesis Prox1 are also vital to the process. Inflammation is a known stimulus for upregulation of Prox1 and thereby lymphangiogenesis [112]. The mechanisms of lymphangiogenesis are generally less understood than those of angiogenesis, due to a lack of specific cellular markers. However, in Prox1 null mice, vasculogenesis and angiogenesis are unaffected, but the lymphatic system does not develop. Therefore, Prox1 may be an effective marker of a subpopulation of endothelial cells that initiate development of the lymph system [113]. Expression of VEGFR-3, the receptor for VEGF-C, coincides with expression of Prox1, and is believed to be activated by the master regulator [106]. Prox1 promotes expression of FGFR-3 in the lymphatic endothelium, indicating a potentially important role of FGF signaling in lymphangiogenesis [114]. Moreover, knockdown of FGFR-3 mRNA inhibited proliferation of lymphatic endothelial cells, supporting this hypothesis. Lyve-1 and podoplanin are two other examples of lymphatic endothelium-specific markers [111].

#### 4.4.6. Arteriogenesis

**Figure 4:**
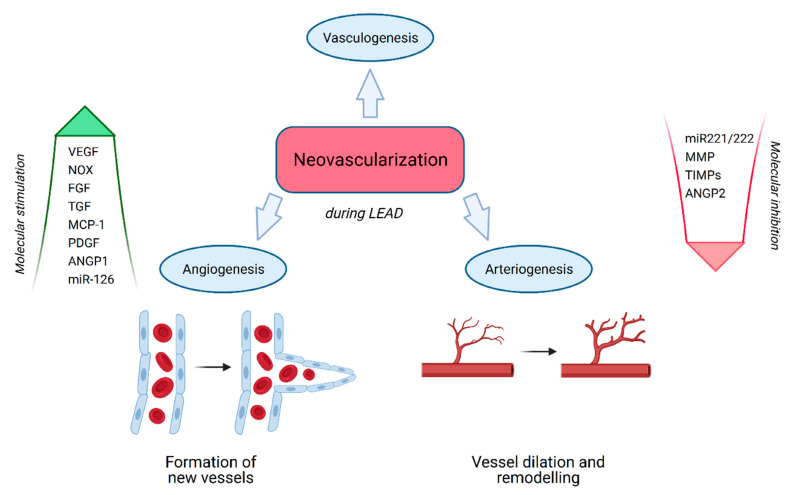
Neovascularization during LEAD. Angiogenesis, described as the process of new capillary formation from pre-existing capillary beds, and arteriogenesis, the process of vessel dilation and remodeling, can be distinguished during LEAD. The most important molecular factors described in this article that can stimulate or inhibit the neovascularization process are presented. Created with BioRender.

Inflammation is an important stressor that induces arteriogenesis, a process leading to the formation of new collateral arteries (Figure 4). Artery occlusion results in increased flow, and thereby stress at the occlusion site. Consequently, fluid shear stress activation of endothelial mechanically-activated calcium TRP channels activates important signaling pathways, resulting in vasodilatation and promoting the upregulation of monocyte chemoattractant protein (MCP-1) and important structural proteins in nearby endothelial and smooth muscle cells [4,115]. One of the most important functions of arteriogenesis is to increase arterial diameter by upregulating the proliferation of smooth muscle cells via MCP-1 recruitment of immune cells and initiation of inflammation [115]. Immune cells recruited to sites of inflammation act as sources of arteriogenic factors such as TGF- β and FGF-2, which facilitate the proliferation of vascular smooth muscle cells to form new arteries [4,75].

Exercise, and in particular structured and supervised exercise programs for patients of peripheral artery disease, is believed to help induce arteriogenesis through increased fluid shear stress activation of ion channels in the endothelium [69]. Further research into the molecular mechanisms of arteriogenesis could reveal drug targets to induce blood vessel growth to treat PAD and LEAD. For example, the inflammatory protein MIDKINE could be vital for upregulating endothelial cell proliferation and signaling following shear stress activation of TRP channels in occlusion sites [116].

## 5. Mechanisms of Neovascularization and Cell-Based Therapies

### 5.1. Endothelial Cells (ECs)

Endothelial cells form a monolayer lining the inside of vessel networks. Endothelial cells are maintained in a quiescent state by factors, such as angiopoietin-1 (ANGP-1) secreted by the surrounding smooth muscle cells and pericytes. High-density lipoproteins promote cell proliferation and migration in the endothelium, and protect endothelial cells from apoptosis [117].

Endothelial cells’ ability to grow in hypoxic environments may be linked to the fact that bi-directional ATP synthase is present on the plasma membrane of these cells, allowing ECs to maintain a high level of intracellular ATP [118]. A 2008 in vitro study using rat hepatocytes demonstrated that Ecto-ATP Synthase expressed on plasma membrane surfaces could be important in extracellular ATP synthesis and reverse ATP hydrolysis, thus regulating the ATP/ADP ratio and maintaining intra/extracellular pH homeostasis [119]. Notably, in ECs, angiostatin has been shown to bind and inhibit the cell surface ATP synthase at the α-subunit, blocking synthesis of extracellular ATP and preventing proliferation and differentiation in hypoxic conditions [118,120]. The nature of plasma membrane ATP synthase and its interactions with angiogenic inhibitors highlights an area in need of research and a possible pharmacological target in disease.

Central to the function of the endothelium is the preservation of the endothelial barrier lining the inside of vessels. EC cell–cell adhesion in the vessel is maintained by adherens junctions composed of vascular endothelial (VE)-cadherin. The ability of the EC barrier to modulate and fine-tune permeability is made possible by the VE-cadherin mediator Rap1, which, in response to appropriate signaling, will modify adherens junctions to allow EC mobility. For example, during angiogenesis, VEGF causes vessel wall leakiness and EC migration to expand existing vessels and create new branches, which is counteracted by the stabilizing effects of ANG-1 [102].

### 5.2. Endothelial Cell Sprouting

VEGF signals in the environment, released from pre-existing vessels provide a path for the developing vessel. In response to VEGF, ECs detach from each other, through alterations in adherens junction complexes and the dissolution of the ECM by plasminogen activators and matrix metalloproteinases [91,121]. This marks the onset of angiogenesis. When sprouting vessels reach the source of the VEGF gradient, the circuit is closed as connections are made with established vessels. There are many components to the developing blood vessel, and three main functionally distinct cell phenotypes. The closest ECs to the source of VEGF, known as tip cells, use the gradient of growth factor to direct migration, and receive the highest levels of VEGF activation. ECs show unique gene expression profiles from those cells further away, which constitute the stalk cells of the developing vessel. By proliferation, stalk cells extend the developing vessel, while phalanx cells play a supportive role during sprouting [121]. VEGF levels activate expression and release of Dll4 (Notch ligand) by tip cells. Subsequent Notch signaling activation by Dll4 binding Notch receptors in adjacent stalk cells prevents tip-cell activity of these cells. Notch signaling between adjacent ECs is thereby vital to coordinating their actions during sprouting angiogenesis [84]. The directional migration of ECs during sprouting is known as haptotaxis. The initial sprouting results in a string of endothelial cells, which later forms a functional tube, which is then supported by pericytes and smooth muscle cells, marking maturation of the vessel. Angiopoietin and platelet-derived growth factor (PDGF) are two factors involved in attracting the supporting cells to the new vessel after the basement membrane is newly established [91].

### 5.3. Stem Cell Therapy and Angiogenesis

Hematopoietic stem cells derived from bone marrow, show promise in treatment of myocardial infarction by enhancing restoration of heart function, but they are difficult to isolate and not abundant enough to provide a useful source of cells for therapy. Therefore, research focus has shifted in terms of cell-based therapies to plentiful and multipotent cell populations, which can be safely and easily harvested from adipose tissue. These cells additionally contain higher proliferative capacity than bone marrow-derived stem cells, increasing their usefulness in therapy [122].

Mesenchymal stromal cells (MSCs) and adipose-derived stem cells (ASCs) are important cell sources providing therapeutic options for various diseases, including those of the circulatory system. The pro-angiogenic effects on ischemic disease following transplantation of these cell types is key to the future treatment of heart and vessel disease. The secretome of MSCs contains a range of factors that can aid in angiogenesis, including VEGF and other growth factors, miRNAs and lipids [123]. MSCs can enhance angiogenesis both in vitro and in vivo, due to the bioactive molecules contained within vesicles such as exosomes released from these cells [124]. A 2017 study investigating the angiogenic potential of extracellular vesicles (EVs) derived from MSCs showed that these vesicles, upon delivery to sites of injury, could enhance angiogenesis in mice. Western blotting results showed increased levels of VEGF in EVs as compared to MSCs, therefore suggesting that the secretome of MSCs can directly activate VEGFR in mouse endothelial cells. In addition, expression of VEGFR in mouse endothelial cells was significantly increased after treatments with EVs. The delivery of EVs alone as opposed to whole cells is generally a safer option, as tumorigenesis can be a side effect of cell transplantation [125].

In a mouse model, intramuscular injections of ASCs showed effectiveness in enhancing wound healing. Dermal wound healing, blood perfusion to the skin and blood capillary density were all increased due to paracrine factors including angiogenic factors released by ASCs upon transplantation. The positive effects were also explained in part due to ASC differentiation into keratinocytes and endothelial cells, incorporation into capillaries, dermal and epidermal tissue; confirmed by GFP tagging and expression of specific cell markers [126].

In terms of clinical trials in humans, the 2014 ACellDREAM study established the efficacy and safety of autologous ASC treatment via intramuscular injections to treat peripheral artery disease. The patients involved in the study were all determined to have non-revascularizable critical limb ischemia, and, although the scope of the study was limited, wound healing showed improvement in most patients [127].

The 2017 ATHENA trials examined autologous adipose-derived regenerative cell fractions (ADRC) as potential treatments for patients with chronic ischemic cardiomyopathy. The ADRC constituted a heterologous cell population including ASCs, endothelial cells, pericytes, smooth muscle cells and immune cells. Although stopped prematurely, the trial indicated a benefit to symptoms of heart failure and angina in patients treated with cell transplantation, as well as self-reported improvements via patient questionnaires [128].

Additional in vivo and in vitro experiments have been undertaken to highlight the contribution of cellular signaling and extracellular vesicles (EVs) in stem cell therapies, and in particular, induction of angiogenesis. Magnetic field treatment has been proposed as a method of inducing secretion of growth factor-rich EVs from ASCs [129]. In 2016, an in vitro study showed that preconditioning ASCs in endothelial cell differentiation medium was effective. Microvesicles released from these ASCs were isolated and demonstrated to induce angiogenesis in vitro. miRNA levels were enriched in these vesicles, and miR-31 was shown to play a role in the microvesicle-induced angiogenesis. miR-31 is known to augment migration of endothelial cells and is upregulated in ischemic hind limbs and VEGF-treated HUVEC cells. Additionally, the five known target genes of miR-31 code for anti-angiogenic proteins. This study both demonstrated the importance of EVs in cellular therapies, and showed that the hypoxia-inducible factor 1-alpha inhibitor protein in endothelial cells may be a potential target for miR-31 in pro-angiogenic ASC microvesicles [130].

### 5.4. Molecular Association with Traditional Therapies in LEAD

In clinical practice, strategies for the treatment of atherosclerosis focus on the reduction of the risk factors of this pathological condition, lifestyle modification, and surgical revascularization. As discussed previously, the formation of atherosclerotic lesions can be driven by multiple interdependent risk factors. Arterial hypertension, diabetes mellitus, and hypercholesterolemia drastically change the functionality of the capillary vessels. Therefore, the proper pharmacological treatment strategy of these pathologies is essential. Current therapeutic options of LEAD focus on improving limb symptoms or preventing amputation. Smoking cessation combined with regular exercise provides the most noticeable improvement. However, exercise therapy is impossible in patients with CLTI. Several studies have shown that statins significantly improve not only CV prognosis of patients with LEAD, but also walking distance capacity [131,132,133]. Other pharmacological agents, such as prostanoids (prostaglandin I2 and E1), cilostazol, and naftidrofuryl also have favorable effects on walking distance and leg function [134,135].

LEAD symptoms may be the result of not only an obstructive vascular process but also impaired neovascularization response. Supervised physical exercise (PE), a natural stimulus for vascular remodeling, plays a crucial role in the pathophysiology of PAD both in prevention and treatment, and is currently considered to be a potential therapeutic option in chronic LEAD patients under conservative treatment. Exercise therapy can influence the vascular system supply to the ischemic limb by promoting both angiogenesis and arteriogenesis [33]. An increase of angiogenic and arteriogenic factors, such as VEGF and HIF-1 alpha, was observed after regular exercise [70].

Stem cell and angiogenic gene therapy in the treatment of LEAD are still being investigated. However, current data has insufficient evidence in favor of these treatment alternatives [99,136,137]. The development of therapeutic angiogenesis is based on the use of angiogenic factors or stem cells to promote revascularization and remodeling of collaterals to reduce ischemia and prevent amputation. Several trials have reported relief of ischemic symptoms, functional improvement, and prevention of amputation. Nevertheless, others have failed to confirm this early promise of efficacy [138,139,140].

## 6. Conclusions

It is important to consider the molecular basis of vascular diseases including lower extremity arterial disease because it is a major health issue. The mechanism of the neovascularization process that occurs during LEAD has been described. A group of environmental and molecular factors influencing the angiogenesis and arteriogenesis processes taking place in the disease state is presented. The description of prospective therapeutic methods using molecular mechanisms indicates that the precise knowledge of the molecular basis of neovascularization may be a very promising therapeutic target in the treatment of LEAD.
